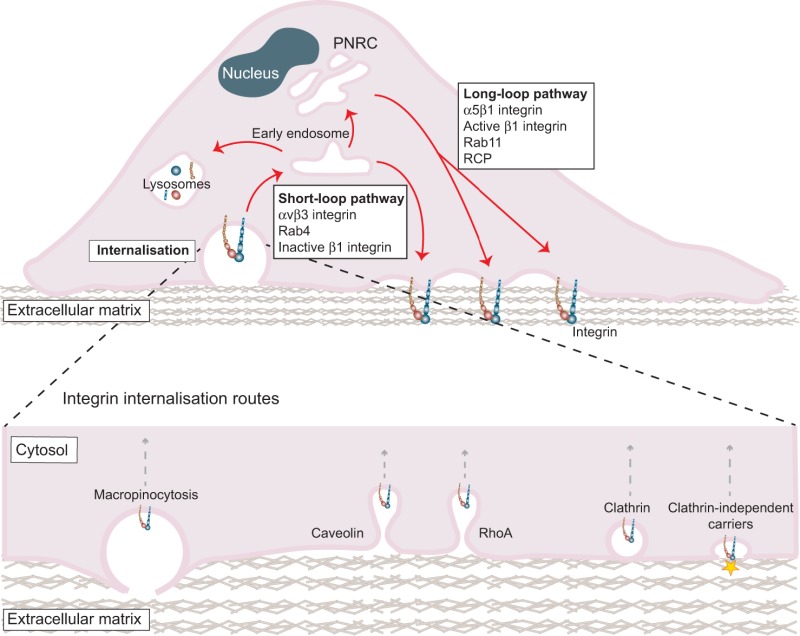# Integrin traffic – the update

**DOI:** 10.1242/jcs.161653

**Published:** 2015-03-01

**Authors:** Nicola De Franceschi, Hellyeh Hamidi, Jonna Alanko, Pranshu Sahgal, Johanna Ivaska

**Affiliations:** Turku Centre for Biotechnology, University of Turku, Turku 20521, Finland

**Keywords:** Integrin, Trafficking, Rab GTPases, Migration, Invasion, Signalling crosstalk

## Abstract

Integrins are a family of transmembrane cell surface molecules that constitute the principal adhesion receptors for the extracellular matrix (ECM) and are indispensable for the existence of multicellular organisms. In vertebrates, 24 different integrin heterodimers exist with differing substrate specificity and tissue expression. Integrin–extracellular-ligand interaction provides a physical anchor for the cell and triggers a vast array of intracellular signalling events that determine cell fate. Dynamic remodelling of adhesions, through rapid endocytic and exocytic trafficking of integrin receptors, is an important mechanism employed by cells to regulate integrin–ECM interactions, and thus cellular signalling, during processes such as cell migration, invasion and cytokinesis. The initial concept of integrin traffic as a means to translocate adhesion receptors within the cell has now been expanded with the growing appreciation that traffic is intimately linked to the cell signalling apparatus. Furthermore, endosomal pathways are emerging as crucial regulators of integrin stability and expression in cells. Thus, integrin traffic is relevant in a number of pathological conditions, especially in cancer. Nearly a decade ago we wrote a Commentary in *Journal of Cell Science* entitled ‘Integrin traffic’. With the advances in the field, we felt it would be appropriate to provide the growing number of researchers interested in integrin traffic with an update.

## Introduction

Integrins are among the most abundant cell surface receptors and are expressed in all cell types apart from erythrocytes; they constitute the principal adhesion receptors for the extracellular matrix (ECM). Integrins were originally named to denote their role as integral membrane complexes linking the ECM to the actin cytoskeleton. However, it is now clear that integrins alone, or in combination with other cell surface receptors, mediate many key intracellular signals and are indispensable for the existence of multicellular organisms. As such, tight regulation of integrin signalling is paramount for normal physiological function, and misregulated integrin activity is associated with many pathological conditions including cancer. The regulation of integrin function can be achieved on several levels, including ligand engagement and binding of intracellular proteins. Endocytic trafficking of integrins offers an important complementary mechanism for regulating integrin–ECM adhesion turnover, and thus integrin signalling, by tightly controlling specific integrin heterodimer availability at the cell surface. With increasing interest on the biological relevance of integrin traffic, the number of adaptor and signalling proteins and cellular machineries identified as key regulators of this pathway is rapidly expanding and thus reveals the complexity of integrin traffic regulation and the close connection with the cytoskeletal and signalling apparatus of a cell. Importantly, conceptual progress in the field has identified well-known cancer oncogenes and mutations as being crucial regulators of integrin traffic and, therefore, cell invasion and metastasis. Mounting data highlights how integrin trafficking is deeply wired into the cytoskeletal and signalling apparatus of a cell and how integrin trafficking is tightly and spatially regulated in the cell. In this Commentary, we will describe the newest findings in the regulation of integrin traffic. In addition, we have compiled several examples of the role of integrin traffic in different pathophysiological conditions, with a particular focus on cancer metastasis (Table 1).

**Table 1.**
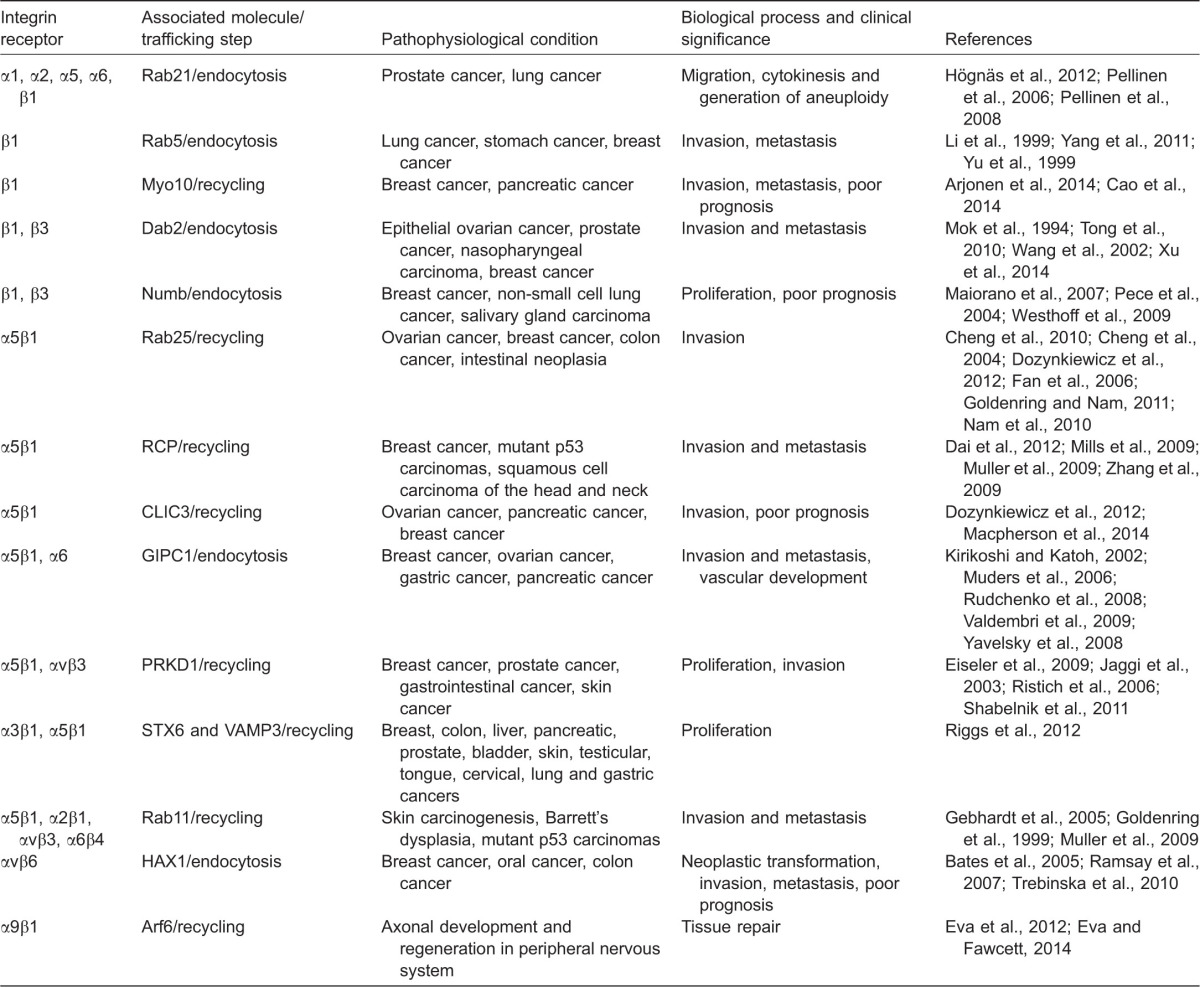
Integrin traffic pathways and associated pathophysiological conditions in humans

## Canonical integrin trafficking

In the classical model of integrin traffic – which still holds true to a large extent – cell surface integrins are constitutively endocytosed through clathrin- or caveolin-mediated routes and undergo endosomal sorting that determines degradation or recycling of the receptor. The role of integrin degradation was neglected for a long time due to the overall long half-life of integrins. However, several studies now suggest that trafficking through the degradative pathway is coupled to integrin activity and receptor dynamics in migrating cells (examples are discussed in the following sections), even though most integrin receptors appear not to be degraded. Instead, integrins are recycled back to the membrane by one of two spatially and temporally distinct mechanisms (Caswell and Norman, 2006; Morgan et al., 2009; Scita and Di Fiore, 2010) to provide the cell with a constant fresh pool of integrins to engage the matrix and generate new adhesions (Box 1). With emerging new evidence, this picture is rapidly developing in complexity, and cell-type- and context-dependent variations to the general dogma have been documented. Reports from recent years have demonstrated integrin recycling from late endosomes (Dozynkiewicz et al., 2012) or directly from tubular Arf6-containing endosomes that bypass the early endosome compartments (Chen et al., 2014). New routes of integrin endocytosis include macropinocytosis from circular dorsal ruffles (CDRs) triggered by growth factor receptor signalling (Gu et al., 2011) and internalisation by clathrin-independent carriers (Lakshminarayan et al., 2014) (Box 1). It is evident that various distinct and previously unappreciated cellular components are involved in the regulation of integrin traffic and that, in most cases, these are pathways that are highly dependent on the integrin heterodimer and cell type, as well as on stimuli and the matrix (Bridgewater et al., 2012; Caswell and Norman, 2006). Importantly, the route of integrin internalisation and recycling has been shown to dictate the ability of these receptors to promote two-dimensional (2D) and three-dimensional (3D) cell migration, and as discussed below, this requires the integration of multiple signals from matrix ligation, GTPase activity and receptor crosstalk to altered membrane dynamics and the cytoskeletal machinery.

## Integrin–ECM adhesions and integrin traffic

Integrin–ECM adhesions can be classified into several subtypes based on morphology, protein composition and stability (reviewed in Valdembri and Serini, 2012). Clustering of integrins in newly formed nascent adhesions leads to the recruitment of adaptor and signalling proteins and the formation of focal complexes. The maturation of focal complexes into focal adhesions (FAs) serves to anchor the ECM to the actin cytoskeleton and the centripetal translocation of the α5β1 integrin heterodimer towards the cell body leads to the formation of very long and stable tensin-rich fibrillar adhesions. Invadopodia and filopodia represent specialized actin-rich matrix contacts that also contain integrins. Invadopodia are typically confined to invasive cells (Linder et al., 2011) and are sites of active ECM degradation by the transmembrane type 1 matrix metalloproteinase (MT1-MMP, also known as MMP14). Filopodia are integrin-dependent structures that have been recently implicated in cancer cell invasion (Arjonen et al., 2014; Machesky and Li, 2010). Although invadopodia and filopodia share some similarities and common features, invadopodia-mediated cell invasion appears to be dependent on active MT1-MMP recycling (Monteiro et al., 2013), whereas filopodia-driven cell invasion occurs as a consequence of increased integrin recycling (Arjonen et al., 2014). Intriguingly, integrins and MT1-MMP can be recycled along the same pathway (Dozynkiewicz et al., 2012; Macpherson et al., 2014); in particular, β3-integrin–MT1-MMP co-trafficking along a Rab4 recycling pathway has been shown to be necessary for hepatocyte growth factor (HGF)- and Rab5-dependent induction of invadosomes, resulting in cell invasion and matrix degradation (Frittoli et al., 2014). Whether, a similar recycling pathway is implemented in filopodia to promote cell invasion remains to be investigated.

### ECM in integrin traffic

Processing of ECM molecules and/or complete detachment of integrins from their substrates, to allow receptor endocytosis, is a necessary step in the initial stages of integrin traffic. The presence of extracellular molecules in endocytic vesicles supports co-endocytosis of integrins with matrix ligands. For example, fibronectin, a fundamental component of the ECM, is endocytosed with β1 integrins in a caveolin-1-dependent manner and is targeted for lysosomal degradation (Sottile and Chandler, 2005). However, given the large size of fibronectin fibrils, integrin–fibronectin co-endocytosis would not be feasible without proteolytic cleavage of fibronectin fibrils. Therefore, the size of detachable ECM components, regulated by MT1-MMP proteolysis, is crucial for directing α5β1 integrin endocytosis (Shi and Sottile, 2011). In a low-fibronectin ECM environment, the endocytosed fibronectin is re-secreted from late endosomes and lysosomes, possibly together with integrins (Dozynkiewicz et al., 2012), to promote cell migration (Sung et al., 2011). These findings, together with observations that ECM degradation, previously thought to be restricted to invadosomes, also occurs in FAs (Stehbens et al., 2014; Wang and McNiven, 2012), suggest that matrix turnover and integrin traffic can be coupled on multiple levels.

### Integrin traffic and cell–ECM adhesion turnover

The dynamic assembly and disassembly of integrin–ECM adhesions is crucial for the stabilisation of membrane protrusions and the application of tension to the ECM and retraction of the cell rear during cell migration, with integrin recycling being central to the regulation of this process of dynamic adhesion turnover (Caswell et al., 2009). The different adhesion subtypes, mentioned above, exhibit differing turnover rates, suggesting that alternative integrin trafficking routes are employed during the assembly and disassembly processes. Although the exact nature of adhesion-type-specific trafficking is currently not well understood, nevertheless some specific examples have been described. In endothelial cells, Ras and Rab interactor 2 (RIN2) regulates integrin endocytosis from nascent adhesions (Sandri et al., 2012), whereas an endocytic complex consisting of the adaptor GAIP interacting protein C-terminus member 1 (GIPC1) and neuropilin-1 (Nrp1; a cell surface receptor) specifically drives α5β1 integrin endocytosis from fibrillar adhesions (Valdembri et al., 2009).

FA disassembly has been suggested, under some specific conditions, to be linked to endocytosis of ligand-bound active integrins. Proteins such as dynamin-2, focal adhesion kinase (FAK, also known as PTK2), clathrin, disabled-2 (Dab2) and AP2, as well as microtubules, have been shown to regulate FA turnover (Chao and Kunz, 2009; Ezratty et al., 2005; Ezratty et al., 2009). The targeting of these proteins to adhesion sites is fundamentally dependent on the spatially restricted production of phosphatidylinositol 4,5-bisphosphate (PtdIns4,5*P*_2_) at the plasma membrane by type I phosphatidylinositol phosphate kinases (PIPKI) in response to integrin–ECM adhesion (reviewed in Ling et al., 2006). Three PIPKI isoforms (encoded by *PIP5K1A*, *PIP5K1B* and *PIP5K1C* in humans) and multiple splice variants exist in mammalian cells, and two of these have been implicated in FA turnover. PIPKIβ has been shown to mediate the endocytosis of active β1 integrin from zyxin-positive FAs and therefore drive FA disassembly (Chao et al., 2010). Depletion of PIPKIβ blocks clathrin assembly at adhesion plaques, prevents complex formation between dynamin 2 and FAK, and impairs cell migration. Interestingly, in contrast to PIPKIβ, PIPKIγ661 is implicated in FA assembly (Ling et al., 2002). This PIPKI variant regulates the recruitment of talin and vinculin to FAs by local production of PtdIns4,5*P*_2_ (Legate et al., 2011). Thus, functionally distinct pools of PtdIns4,5*P*_2_ produced by PIPKIβ or PIPKIγ661 can function as specific platforms for the recruitment of proteins involved in FA assembly or disassembly. How the activity of these two PIPKI isoforms is coordinated and targeted specifically to FA sites is not yet clear, however post-translational modifications of PIPKI C-terminal domains have been implicated in this process (Chao et al., 2010; Ling et al., 2002).

In mammalian cells, cholesterol, in the form of low-density lipoprotein (LDL), is recruited to FAs from late endosomes in an acid lipase, NPC1- (Niemann-Pick C1 protein) and Rab8a-dependent fashion, resulting in increased number, size and turnover of FAs, and consequently enhanced cancer cell migration (Kanerva et al., 2013). Inhibition of cholesterol levels in the trans-Golgi network triggers syntaxin-6 accumulation into Rab11-positive recycling endosomes and adversely affects αvβ3 and α5β1 integrin recycling and cell migration (Reverter et al., 2014). A role for syntaxin-6 in α3β1 and α5β1 integrin trafficking has also been demonstrated in chemotactic cancer and endothelial cells, respectively (Riggs et al., 2012; Tiwari et al., 2011). Importantly, these studies identify a new integrin recycling step that traverses the trans-Golgi network and highlight an intriguing role for the Golgi and cholesterol traffic in controlling FA dynamics. The degree of cholesterol-mediated effects on FA turnover is likely to be dependent on the rate of cholesterol uptake and the integration of cholesterol into different membrane compartments.

## The cell cytoskeleton and integrin traffic

Integrin engagement by extracellular ligands leads to the initiation of signals that impinge on the organisation of the cell cytoskeleton. Different components of the cellular cytoskeleton – actin, intermediate filaments and microtubules – and their associated molecular motors, in turn, have been implicated in the regulation of adhesion turnover through direct or indirect modulation of integrin endocytosis and/or recycling.

### Microtubules in integrin traffic

**Fig. 1.**
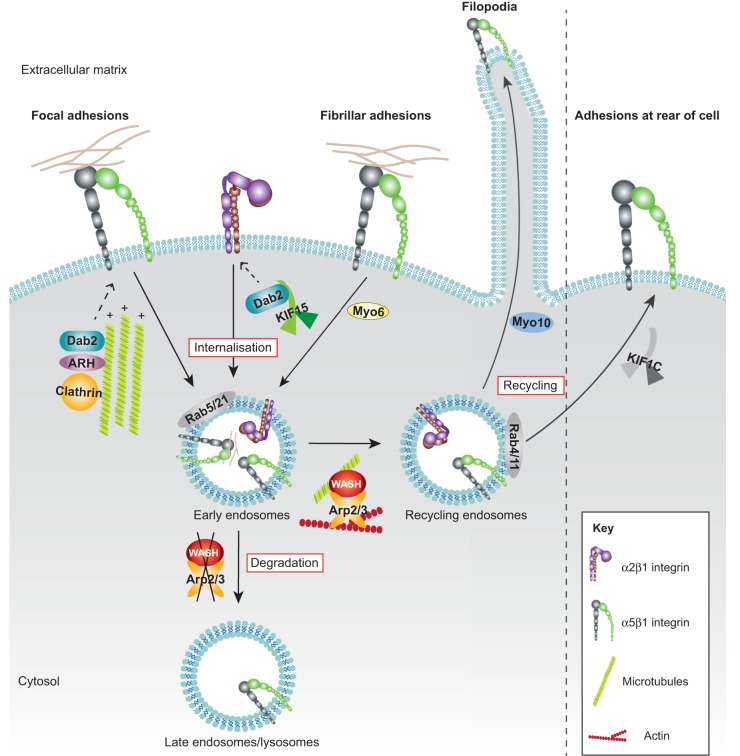
**Role of cytoskeletal proteins in integrin traffic.** Internalisation: clathrin-dependent endocytosis of integrins from FAs is promoted by acetylated microtubules and adaptors, such as Dab2 and ARH. The microtubule motor kinesin KIF15 promotes endocytosis of inactive integrins by delivering Dab2 to these receptors, whereas Myo6, an actin motor, mediates internalisation of α5β1 integrins from fibrillar adhesions. Recycling: in the early endosome compartment, WASH-mediated recruitment of Arp2/3 and subsequent Arp2/3-mediated actin reorganisation drives integrin traffic to recycling endosomes. Here, several mechanisms support integrin recycling back to the plasma membrane. For example, KIF1C promotes α5β1 recycling towards the rear of migrating cells for FA maturation, and Myo10 traffics integrins to filopodia tips. Degradation: in the absence of WASH, integrins are rerouted towards degradation.

Directed intracellular vesicle movement is guided by polarized microtubules and the microtubule-based motor proteins dyneins and kinesins. Microtubules trigger FA disassembly by the local dissolution of adhesions, following physical contact with and delivery of clathrin and two adaptor molecules, autosomal recessive hypercholesterolemia (ARH, also known as LDLRAP1) and Dab2, to the targeted integrin–ECM adhesion site (Ezratty et al., 2005; Ezratty et al., 2009; Kaverina et al., 1999). Although microtubules are not absolutely required for FA disassembly or for clathrin transport to the plasma membrane, nevertheless they increase the rate of FA turnover (Ezratty et al., 2009; Kaverina et al., 2002) (Fig. 1). Very recently, microtubule-mediated delivery of the mitogen-activated protein kinase (MAPK) kinase kinase kinase 4 (MAP4K4) to FAs through its interaction with end-binding 2 (EB2, also known as MAPRE2) protein has been shown to enhance FA dissolution through an Arf6-dependent mechanism (Yue et al., 2014). Microtubule-associated cytoplasmic linker associated proteins 1 and 2 (CLASP1 and CLASP2) have been shown to tether microtubules to FAs, thereby triggering FA disassembly and turnover through the local exocytosis of metalloproteinases. Intriguingly, the accumulation of CLASPs in FAs has been suggested to occur independently of microtubules and to be guided by a signal that is located at FAs (Stehbens et al., 2014). Localisation of α-tubulin acetyltransferase 1 (αTAT1) at paxillin-rich adhesions and its interaction with AP2, present in clathrin-coated pits, has been shown to promote microtubule stabilization at the leading edge, potentially enhancing microtubule-mediated FA turnover (Montagnac et al., 2013).

The microtubule-dependent delivery of cargos, such as organelles and vesicles, is promoted by microtubule motor proteins such as kinesins. In terms of integrin traffic, the kinesin KIF1C directs the recycling of endocytosed α5β1 integrin to the rear of migrating cells (Theisen et al., 2012) (Fig. 1), highlighting a new functional role for FAs at the trailing edge. Here, KIF1C-dependent delivery of integrins is necessary to allow rear FAs to mature and to resist the actin traction force in a process described as ‘rear steering’. In this model, a well-anchored cell tail maintains directional cell migration by assigning a defined polarity to the cell. In contrast, tail retraction leads to loss of cell polarity and re-establishment of a new cell axis (Theisen et al., 2012). KIF15, another kinesin motor, was recently identified in a fluorescence-microscopy-based RNA interference screen as a new regulator of inactive α2β1 integrin traffic (Eskova et al., 2014). However, unlike KIF1C, KIF15 promotes α2β1 integrin endocytosis through the transport of Dab2 to the plasma membrane to engage the α2 integrin tail (Fig. 1). KIF15-triggered endocytosis appears to be integrin specific because Dab2 has not been associated with trafficking of other receptors (Maurer and Cooper, 2006).

### Actin and integrin traffic

In yeast, actin has a fundamental role in vesicle trafficking and endocytosis (Kaksonen et al., 2006). Although actin is dispensable for the process of endocytosis in higher eukaryotes, several studies have indicated an important regulatory role for actin in integrin endosomal traffic particularly on endosomes. Actin-related protein (Arp)2/3, a multiprotein complex and actin nucleator, promotes the assembly of branched F-actin networks at the plasma membrane and on endosomes (Goley and Welch, 2006; Kaksonen et al., 2006). Activation of Arp2/3 is mediated by nucleation-promoting factors such as members of the Wiskott–Aldrich syndrome protein (WASP) family, all of which function in distinct subcellular locations (reviewed in Takenawa and Suetsugu, 2007). WASH (WASP and SCAR homologue, of which there are two isoforms, WASH1 and WASH2) recruits Arp2/3 to early endosomes, where it promotes Arp2/3-dependent actin polymerization, which is necessary for α5β1 integrin recycling (Duleh and Welch, 2012; Zech et al., 2011). WASH depletion reduces integrin recycling from perinuclear early endosome antigen-1 (EEA1)-containing early endosomes, without influencing integrin endocytosis. As a consequence, α5β1 integrin accumulates in CD63-positive (a marker of late endosomes and lysosomes) vesicles (Fig. 1). In ovarian carcinoma cells, WASH is important for invasion into fibronectin-rich matrices and for migration on cell-derived matrices but is dispensable for 2D migration (Zech et al., 2011). However, in fibroblasts, WASH depletion disrupts α5 integrin localization to FAs, decreases FA number and impairs adhesion and migration on fibronectin (Duleh and Welch, 2012). Thus, the requirement for WASH-mediated integrin endocytosis for cell migration on 2D substrates appears to be cell-type-specific or perhaps even cancer specific. Other roles for the actin machinery in integrin traffic include myosin-X (Myo10)-dependent regulation of integrin delivery to filopodia tips (Zhang et al., 2004) to drive cell migration, invasion and metastasis (Arjonen et al., 2014) and myosin-VI (Myo6)- and Rab5-mediated endocytosis of active α5β1 integrin (Valdembri et al., 2009) (Fig. 1).

Taken together, it is clear that the cytoskeletal machinery is crucial for the regulation of integrin traffic. As integrin–ECM engagement at the plasma membrane generates signals that regulate cytoskeletal dynamics in cells, the role of the cytoskeleton in integrin traffic is deeply connected with integrin activity and function.

## Trafficking of active and inactive integrin heterodimers

**Fig. 2.**
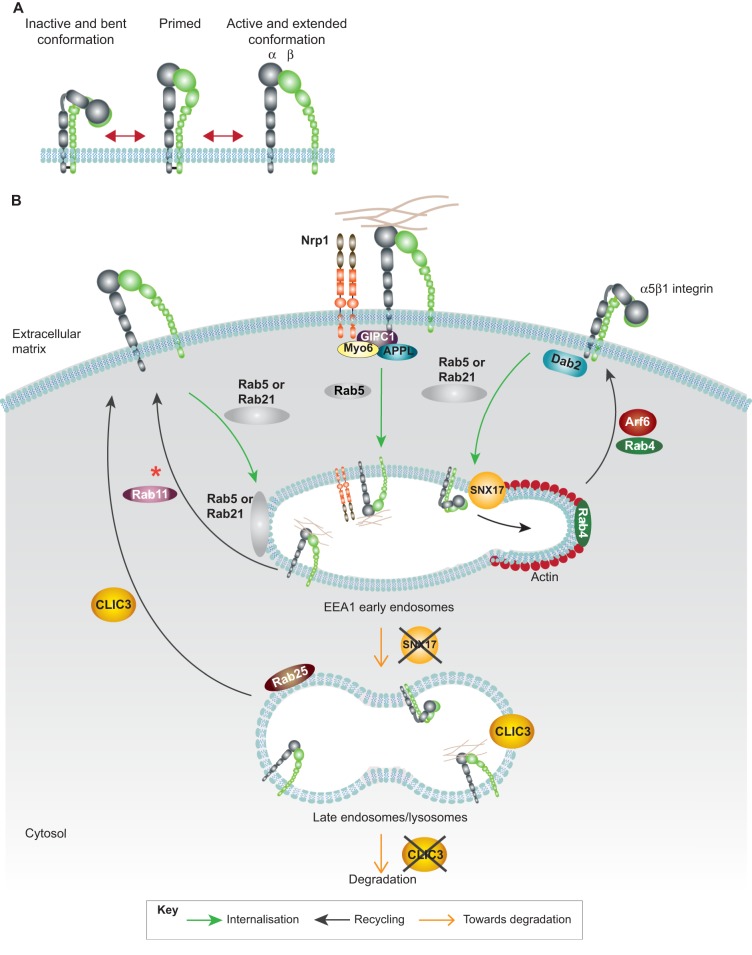
**Trafficking of active and inactive integrin heterodimers.** (A) Stepwise model of integrin activation through a conformational switch. (B) Schematic representation of the trafficking routes of active and inactive integrin heterodimers. Internalisation: at the plasma membrane, both active and inactive integrins are endocytosed to early endosomes in a Rab5- or Rab21-dependent fashion. Dab2 acts as an adaptor for endocytosis of inactive (unengaged) integrins, whereas an Nrp1–GIPC1–Myo6–APPL module mediates endocytosis of active α5β1 integrin from fibrillar adhesions. Recycling: inactive β1 integrins are rapidly recycled to Arf6-positive protrusions in a Rab4-dependent manner, whereas active receptors are trafficked through the Rab11 long-loop pathway (the red asterisk indicates recycling from PNRC – omitted here for simplicity). Degradation: in early endosomes, SNX17 binding to the cytoplasmic domain of inactive β-integrin promotes recycling of the receptor over degradation. Similarly, in the late endosome and lysosome compartment, CLIC3-mediated recycling prevents degradation of the active integrin receptor.

Integrin activity is tightly regulated by intracellular adaptors and extracellular factors that lead to conformational changes in the α-integrin–β-integrin heterodimer. Integrins can exist in either (1) an inactive, bent conformation with a closed head-piece and low affinity for ECM ligands, (2) a primed, extended conformation with a closed head-piece and low affinity for ligand, or (3) an active, extended conformation with an open head-piece and high affinity for ECM ligands (Fig. 2A; reviewed in Askari et al., 2009; Luo et al., 2007). In addition to differing ligand-binding affinity and downstream signalling at the plasma membrane, active and inactive integrin conformers can be trafficked through distinct routes.

Using antibodies specifically recognising integrin activation states (Byron et al., 2009), active and inactive β1 integrins have been shown to be endocytosed through the same clathrin- and dynamin-dependent routes to Rab5- or Rab21-positive EEA1-containing early endosomes in PC-3, MDA-MB-231 and NCI-H460 cells (Fig. 2B; Arjonen et al., 2012). From here, active β1 is recycled through the long Rab11-dependent recycling loop, whereas the inactive β1 receptor is rapidly recycled in an actin- and Rab4-dependent manner to Arf6-positive protrusions at the plasma membrane (Fig. 2B). Consequently, the net endocytosis rate of active β1 integrin is relatively high compared to its inactive conformer and, therefore, in the steady-state situation, inactive β1 is mostly localized at the plasma membrane, whereas active β1 integrin appears to be more cytoplasmic. This localisation is most likely linked to the observation that ligand-bound active integrins traffic to late endosomes and lysosomes, either for degradation (Lobert and Stenmark, 2012) or for ligand detachment, in an increasingly acidic environment, to allow recycling of unbound free integrins back to the plasma membrane. In line with this, active β1 integrin appears to recycle slowly (possibly owing to the requirement for ligand dissociation prior to recycling) and the active, but not the inactive receptor, has been observed in Rab7-positive late endosomes (Arjonen et al., 2012). However, a fascinating study now shows that integrins can be recycled back to the plasma membrane directly from lysosomes while still in their active conformation (Dozynkiewicz et al., 2012). This process, which occurs specifically in cancer cells, requires the concerted activities of Rab25 and chloride intracellular channel protein 3 (CLIC3). Rab25 directs ligand-bound active α5β1 integrins to late endosomes and lysosomes, where, instead of degradation, the receptors undergo retrograde transport to the plasma membrane at the rear of cancer cells in a CLIC3-dependent pathway (Dozynkiewicz et al., 2012). Conversely, in the absence of CLIC3, ligand-bound integrins are directed towards degradation (Fig. 2B). Interestingly, in that study, α5β1 integrin traffic was not dependent on CLIC3 in the absence of exogenously added fibronectin, indicating a role for CLIC3 specifically in the recycling of active ligand-engaged integrins to the back of invading cells. This process was implicated in tail retraction during cell migration on cell-derived matrices and in cell invasion in 3D organotypic microenvironments (Dozynkiewicz et al., 2012). This reveals an intriguing, apparently highly context-dependent role, for α5β1 recycling in the dynamics of the cell rear. As described previously, KIF1C delivery of α5β1 integrin to the cell rear stabilizes FAs, inhibits rear detachment and guides directional migration in a 2D environment (Theisen et al., 2012), whereas, in 3D or on cell-derived matrices, CLIC-3-dependent α5β1 integrin recycling to the cell rear facilitates dissociation of the retracting tail. In addition to roles in integrin recycling, CLIC3 has been associated with cell invasion and poor prognosis in oestrogen receptor (ER)-negative breast cancer independently of Rab25 by directing the recycling of the pro-invasive MT1-MMP from late endosomes to the plasma membrane (Macpherson et al., 2014).

## Small GTPases govern integrin traffic

Small GTPases are important signalling molecules that cycle between an active GTP-loaded form, capable of associating with effectors, and an inactive GDP-loaded form. The switch between the two activation states is primarily mediated by guanine-nucleotide-exchange factors (GEFs) and GTPase-activating proteins (GAPs). Among the Ras superfamily of small GTPases, members of the Rho, Rab and Arf families have been implicated in integrin traffic. The Rho GTPases (Rho, Rac and Cdc42) impact on integrin trafficking by modulating actin cytoskeleton dynamics (Ridley, 2006). The Rab and Arf family of small GTPases have a more dominate role in the regulation of membrane dynamics and receptor trafficking and will be discussed below.

### Rab GTPases and integrin traffic

**Fig. 3.**
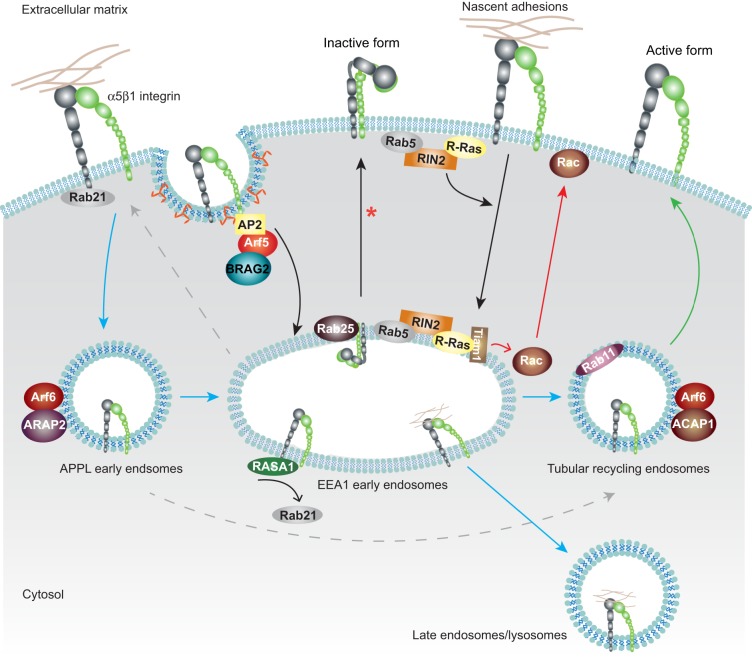
**Role of small GTPases in integrin traffic.** Rab and Arf GTPase family members and their GTPase regulators are intimately involved in different steps of trafficking pathways. Internalization: integrin endocytosis to early endosomes can be triggered by both Rab5 and Rab21, regardless of integrin activation status. Other reported pathways of integrin internalization involve the activation of Arf5 facilitated by a BRAG2–clathrin–AP2 complex at clathrin-coated pits (dependent on PtdIns4,5*P*_2_), or the activation of R-Ras and the formation of an R-Ras–RIN2–Rab5 complex at the plasma membrane downstream of integrin–ECM signalling. In early endosomes, this complex also activates Rac1, through the Rac1 GEF Tiam1, ultimately promoting Rac1 translocation to the plasma membrane and the assembly of new nascent adhesions at lamellipodia (Rac1 activation and translocation is represented by red arrows). Recycling: in EEA1-containing early endosomes, RASA1 outcompetes Rab21 for binding to the β-integrin subunit and drives β1 integrin recycling. Rab25 interacts directly with α5β1 on endosomes to facilitate receptor recycling. Arf6-dependent β1 integrin recycling is regulated by the Arf6 GAPs ARAP2 and ACAP1 which localise to different Arf6-positive endosome compartments. ARAP2 promotes the transition of the integrin receptor from APPL-containing endosomes to EEA1-containing early endosomes and towards recycling compartments and/or degradation (blue arrows). In addition, ARAP2 might prevent the direct recycling of integrins from the early endosomes to the tubular recycling compartments or to the membrane (grey arrows). ACAP1 has been implicated in rapid β1 integrin recycling from Arf6-positive tubular endosomes (green arrow). The red asterisk indicates integrin traffic from recycling endosomes.

Rab proteins constitute the largest family of Ras-related small GTPase molecules that facilitate docking and fusion of transport vesicles (Zerial and McBride, 2001). The Rab family members are compartmentalised into specific endosomal membranes, suggesting that there is a unique function for each GTPase in the recycling pathways (Sönnichsen et al., 2000). Integrin endocytosis from the plasma membrane to the early endosomes is regulated by Rab5 and Rab21. Rab21 interacts with integrins directly (Pellinen and Ivaska, 2006) through the conserved membrane-proximal WKLGFFKR sequence found in the majority of the integrin α-tails (Hynes, 2002) and mediates β1 integrin endocytosis to EEA1-containing early endosomes (Mai et al., 2011). Here, p120RasGAP (RASA1) competes with Rab21 for the same binding sites within the integrin cytoplasmic domain. Displacement of Rab21 by RASA1 drives β1 integrin recycling from EEA1-containing endosomes back to the plasma membrane and is important for directional cell motility (Mai et al., 2011) (Fig. 3). Rab25 also interacts directly with α5β1 integrins through the β1-subunit cytoplasmic domain. However, unlike Rab21, Rab25 interacts with integrin on endosomes and promotes differential recycling of integrins based on receptor activation status. Inactive integrin receptors undergo locally restricted recycling in extended protrusions of invading cells to drive invasion, whereas active receptors might be targeted for degradation in late endosomes (Bridgewater et al., 2012; Caswell et al., 2007) (Figs 2 and 3).

To date, the GEFs and GAPs that regulate Rab21 and Rab25 activities in integrin traffic remain elusive. In contrast, there is some evidence for specific Rab5 GEFs and GAPs in modulation of integrin-specific endocytic routes and integrin signalling. RIN2, a Rab5-specific GEF, together with R-Ras has been implicated in β1 integrin endocytosis (Sandri et al., 2012). R-Ras, which is mainly expressed in endothelial and vascular cells, is activated by ECM-induced integrin ligation at the plasma membrane and recruits RIN2 to nascent adhesions in lamellipodia. R-Ras inhibits RIN2 GEF-activity towards Rab5, converting RIN2 into an adaptor with increased affinity for GTP-loaded Rab5, and targets Rab5 to the plasma membrane to promote the endocytosis of active, ligand-bound β1 integrin together with the R-Ras–RIN2–Rab5 complex. In endosomes, R-Ras activates Rac1 through the Rac1 GEF Tiam1 (T-lymphoma invasion and metastasis-inducing protein 1), leading to Arf6-mediated transport of Rac1-GTP to the plasma membrane (Holly et al., 2005; Radhakrishna et al., 1999; Sandri et al., 2012) to promote actin polymerization and assembly of nascent adhesions at the lamellipodia. This further triggers R-Ras activation, thus creating a positive feedback mechanism to promote cell migration (Fig. 3). RN-tre (also known as USP6NL) represents a Rab5 GAP that localizes to FAs and Rab5-positive endosomes and inhibits endocytosis of β1, but not of β3 integrin, to regulate FA turnover and chemotactic cell migration (Palamidessi et al., 2013).

### Arf GTPases and integrin traffic

Arfs represent the smallest family of the Ras superfamily of small GTPases and key regulators of membrane dynamics. Arf6 has an established role in both clathrin-independent integrin internalisation (Sakurai et al., 2010; Yue et al., 2014) and integrin recycling back to the plasma membrane (Dunphy et al., 2006; Morgan et al., 2013; Powelka et al., 2004) to regulate FA dynamics and cell migration (Morgan et al., 2013). In addition, inactive β1 integrin localizes in Arf6-positive protrusions at the plasma membrane (Arjonen et al., 2012). A recent study has demonstrated how Arf6 binding to structurally distinct GAPs can further fine-tune Arf6-dependent integrin trafficking pathways. The Arf6 GAPs ACAP1 and ARAP2 localize to distinct Arf6-positive endosomal compartments and exhibit opposing effects on FA morphology (Chen et al., 2014). ARAP2 binds to the endocytic protein APPL and mediates transport of β1 integrin from the very early APPL-containing endosomes through EEA1-containing early endosomes, whereas ACAP1 is associated with Rab11-containing tubular recycling compartments and faster integrin recycling (Dai et al., 2004) (Fig. 3). All of these trafficking steps appear to be specific for integrin receptors, as depletion of the key mediators of these pathways has no effect on transferrin receptor or epidermal growth factor receptor (EGFR) trafficking (Chen et al., 2014; Moravec et al., 2012).

Another member of the Arf family, Arf5, has recently been implicated specifically in β1 integrin endocytosis (Moravec et al., 2012). BRAG2, a member of the BRAG family (brefeldin-resistant Arf GEFs), is able to activate Arf4, Arf5 and Arf6, but only Arf5 activation has been linked to integrin endocytosis from fibrillar adhesions or FAs (Dunphy et al., 2006; Moravec et al., 2012). BRAG2 is recruited to clathrin-coated pits at the plasma membrane through direct interaction with clathrin and AP2 (Moravec et al., 2012) (Fig. 3) and further interacts with plasma membrane PtdIns4,5*P*_2_, which has been shown to potentiate the GEF activity of BRAG2 (Sakurai et al., 2011). BRAG2-mediated activation of Arf5 at the plasma membrane drives the endocytosis of active α5β1 integrins to early endosomes. Interestingly, the BRAG2-driven endocytic pathway appears to be specific for this heterodimer, as BRAG2 only mediates the recycling, but not the endocytosis, of the αvβ3 integrin heterodimer (Manavski et al., 2014) (Fig. 3).

In addition to GEFs and GAPs, Rab- and Arf-dependent roles in integrin traffic can be further defined by specific downstream effectors (not discussed in this review). Importantly, the precise spatiotemporal regulation of integrin endocytic and excocytic traffic is likely to be highly dependent on the coordinated activities of Rabs, Arfs and their effectors.

## Heterodimer specificity of integrin traffic in cell migration and invasion

### Interactions at the integrin cytoplasmic domain

The cytoplasmic tails of integrins are critical in the translation and coordination of extracellular signals to determine cell fate. As the integrin cytoplasmic domain lacks enzymatic activity, integrin-mediated signalling is initiated by direct recruitment of intracellular proteins to form macromolecular complexes at sites of cell–ECM contact (FAs) (Zaidel-Bar and Geiger, 2010). As such, both α- and β-integrin subunits contain a wealth of overlapping linear motifs in their cytoplasmic domains and the complexity of these molecular codes is becoming more apparent as new binding sites are continuing to be defined.

In the β subunit, the NPxY motifs have been shown to regulate integrin endocytosis by binding to clathrin adaptors Numb (Nishimura and Kaibuchi, 2007) and Dab2 (Teckchandani et al., 2012). During retina morphogenesis, the transmembrane protein Opo (also known as OFCC1) antagonises Numb-dependent integrin endocytosis by directly competing for Numb binding through an integrin-like NPxF motif. Thus, Opo might promote polarized integrin localization to aid in optic cup folding (Bogdanović et al., 2012). This spatial restriction of Numb-mediated integrin traffic has also been linked to efficient cell migration. Phosphorylation of Numb by atypical protein kinase C (aPKC) prevents its association with integrins, the AP2 and Par complex, and recruitment to clathrin-coated structures. Subsequent Numb dephosphorylation, at the leading edge of a migrating cell, promotes localised integrin endocytosis (Nishimura and Kaibuchi, 2007). Dab2 has been implicated both in the turnover of ligand-engaged and ligand-free integrins in different cell types. In HeLa cells, Dab2 regulates β1 integrin endocytosis from the dorsal membrane in 2D microenvironments, to deliver fresh receptors during cell migration (Teckchandani et al., 2012), whereas in fibroblasts, Dab2 plays a role in microtubule-induced FA disassembly (Ezratty et al., 2009). In the endosomal compartment, the sorting nexin family members SNX17 and SNX31 disrupt integrin–kindlin-2 interaction by binding to the same membrane-distal integrin NPxY motif as kindlin-2 and preventing β1 integrin from progressing along the degradative pathway by a yet unknown mechanism (Böttcher et al., 2012; Tseng et al., 2014).

Other less-conserved motifs have been identified on the β-subunit and are potentially involved in heterodimer-specific traffic regulation. Binding of ACAP1 to β1 integrin at cytoplasmic residues 10–15 (which are also found in β3 and β5 integrins) elicits a recycling signal (Bai et al., 2012). In addition, HAX-1 binding to β6 integrin between residues 731 and 758 triggers integrin endocytosis and impacts on cancer cell migration (Ramsay et al., 2007).

On the α-subunit, the importance of the conserved GFFKR motif in integrin traffic is exemplified by the role of Rab21 in endocytosis and, in particular, during cytokinesis (Pellinen et al., 2008). Similar to the β-subunit, the concept of competition equally applies to the α-subunit, where RASA1 competes with Rab21 for the same binding site to promote integrin recycling (Mai et al., 2011).

### Differential heterodimer recycling

**Fig. 4.**
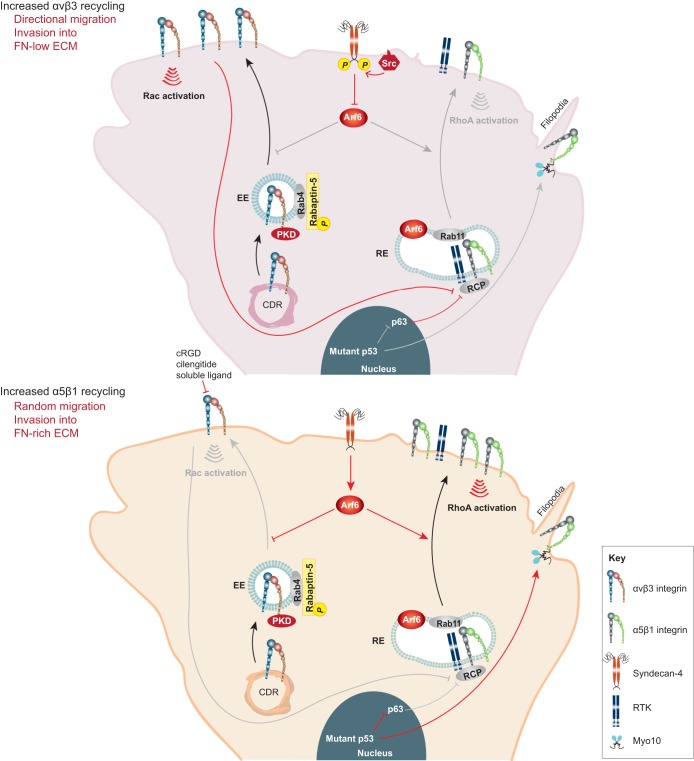
**Effect of reciprocal recycling of α5β1 and αvβ3 integrins on cell invasion.** Top panel: increased αvβ3 recycling. In many cell types, including cancer cells, αvβ3 recycling (e.g. following PDGF stimulation) suppresses the recycling of α5β1 to promote Rac-mediated cell migration in 2D and invasion into low-fibronectin ECM. Bottom panel: increased α5β1 recycling. Manipulating αvβ3 recycling, either by blocking adhesive function (e.g. treatment with cilengitide or cRGD) or indirectly through expression of mutant p53 (found in cancer cells and associated with increased Myo10 expression and filopodia formation) or by manipulating syndecan-4 phosphorylation and/or engagement (important for regulation of Arf6 activity), promotes α5β1 integrin traffic. Increased α5β1 recycling then triggers a RhoA–ROCK-dependent mode of 2D random cell migration, and cell invasion into fibronectin-rich ECM. Red arrows delineate signalling events, whereas black and grey arrows indicate active and inactive endocytic trafficking pathways, respectively. EE, early endosome; FN, fibronectin; LE, late endosome; RE, recycling endosome; RTK, receptor tyrosine kinase.

The 24 functionally distinct integrin heterodimers in mammals have overlapping binding specificities to the ECM. However, binding of a heterodimer to the same ECM ligand can elicit very different biological responses. One of the best examples is the differential receptor recycling and crosstalk of the fibronectin receptors α5β1 and αvβ3. Several studies have demonstrated a mechanism whereby the activity of αvβ3 integrin suppresses recycling of α5β1 integrin (White et al., 2007). In the context of cell migration, αvβ3 integrin recycling supports Rac-dependent lamellipodia formation and directionally persistent cell migration. Conversely, α5β1 integrin recycling induces fast random cell migration through a Rho–ROCK–cofilin pathway (Danen et al., 2005; Morgan et al., 2013; White et al., 2007). When cancer cells migrate invasively through a 3D microenvironment that is rich in fibronectin, increased recycling of α5β1 promotes invasion through the reorganization of the actin cytoskeleton and cell morphology – characterized by the extension of long pseudopods (Caswell and Norman, 2008; Jacquemet et al., 2013; Rainero et al., 2012). The pathways that have been implicated in the switch from αvβ3- to α5β1-mediated recycling are described below and are summarized in Fig. 4.

In some cases, recycling of specific integrin heterodimers has been demonstrated but the basis for the specificity remains to be identified. Rab25 regulates the localized recycling of α5β1 integrin in protrusions of invading cells. However, the interaction is mediated by the β1 cytoplasmic tail (Caswell et al., 2007), which is shared by several different integrin heterodimers, and therefore does not explain the heterodimer specificity of this pathway.

### Role of growth factor signalling, Src and mutant p53 in integrin-heterodimer-specific recycling

One important mechanism that defines the differential recycling of integrin heterodimers is the growth-factor-stimulated delivery of integrins back to the plasma membrane. Under basal conditions, internalized α5β1 and αvβ3 enter the PNRC and are recycled back to the plasma membrane in a Rab11- and PKB/Akt-dependent manner. However, stimulation with platelet-derived growth factor (PDGF), diverts αvβ3 integrin, but not α5β1, through a fast short-loop recycling route that involves Rab4 and the association of αvβ3 with the PKC-related kinase PKD1 (also known as PRKD1) (Woods et al., 2004). PDGF stimulation promotes PKD1-mediated phosphorylation of rabaptin-5 (a Rab5 effector) and the subsequent formation of a rabaptin-5–Rab5–Rab4 complex (Christoforides et al., 2012). This complex triggers delivery of αvβ3 integrin to the leading edge of migrating cells for persistent cell migration and an αvβ3-dependent mode of cell invasion into microenvironments with low fibronectin content (Christoforides et al., 2012). Interestingly, PDGF has also been implicated in integrin endocytosis from CDRs, actin-dependent structures at the dorsal cell surface, by macropinocytosis (Gu et al., 2011), suggesting that context-dependent responses of integrins to growth factors exist. In another study, HGF has been shown to induce rapid β1 integrin endocytosis to drive protease- and integrin-dependent collective cell migration in 3D Matrigel in a process requiring clathrin, RhoA and the clathrin adaptor HIP1 (Mai et al., 2014). HIP1 in complex with the clathrin light-chain has also been shown to be necessary for the recycling of the endocytosed inactive integrin and to promote cell migration in fast-migrating lung cancer cells (Majeed et al., 2014). However, whether growth factor signalling feeds into this trafficking route was not addressed. Nevertheless, there are clear indications that crosstalk between integrins and growth factor receptors functions not only to integrate multiple extracellular signals but also to modify integrin traffic and adhesion dynamics.

Src is a tyrosine kinase implicated in many cancer types. Interestingly, it has also been linked to integrin traffic and migration. Src-mediated phosphorylation of syndecan-4 (a proteoglycan receptor for ECM molecules and growth factors) inhibits Arf6 activation and Arf6-dependent α5β1 integrin recycling, leading to increased FA stabilization and directionally persistent cell migration on cell-derived matrices. In the absence of Src-mediated syndecan-4 phosphorylation, increased α5β1 recycling accelerates FA turnover and inhibits cell migration (Morgan et al., 2013) (Fig. 4). Disruption of syndecan-4 recycling leads to the accumulation of fibroblastic growth factor (FGF) and β1 integrin in syndecan-containing endosomes, suggesting potential co-trafficking of these receptors (Zimmermann et al., 2005).

Differential recycling, which determines the bioavailability of αvβ3 and α5β1 integrins to engage matrix ligands, has also been identified as a crucial mechanism regulating the mode of cancer cell invasion in 3D microenvironments. Integrin association with the Rab-coupling protein [RCP; also known as Rab11 family interacting protein-1 (RAB11FIP1)] determines which heterodimer is recycled back to the membrane (Caswell et al., 2009; Muller et al., 2009). Cancer cell invasion can occur by several mechanisms that are characterized by dramatic changes in cell morphology and actin reorganization that are dependent on the antagonistic activities of Rac1 and RhoA downstream of αvβ3 and α5β1 integrin signalling, respectively. In fibroblasts and cancer cells expressing both αvβ3 and α5β1, RCP is associated with αvβ3 integrin. Gain-of-function mutants of the tumour suppressor p53 or the disruption of αvβ3 function using αvβ3 inhibitors releases β3-associated RCP. RCP then binds to α5β1 integrin and promotes its recycling to phosphatidic-acid-rich membranes at the tip of invasive cell pseudopods (Caswell and Norman, 2008; Muller et al., 2009; Rainero et al., 2012). RCP and α5β1 integrin cooperatively recruit receptor tyrosine kinases, including EGFR1, to further regulate trafficking and downstream signalling on the plasma membrane through the activation of PKB/Akt. PKB phosphorylates RacGAP1 and so triggers its recruitment to IQGAP1 at the invasive front. This pathway downstream of integrin recycling suppresses Rac activity and concomitantly activates RhoA locally in these specific subcellular regions at the cell front. The Rac-to-RhoA switch promotes the extension of pseudopod protrusions and invasive migration into fibronectin-containing matrices (Jacquemet et al., 2013). These protrusions contain actin spikes that resemble filopodia. However, how increased α5β1 recycling and downstream Rho activation influences filopodia formation remains to be determined. Interestingly, mutant p53 also induces expression of the filopodial protein myosin-X, leading to enhanced filopodia formation and cell invasion in breast and pancreatic cancer (Arjonen et al., 2014). This is linked to the ability of myosin-X to transport integrin β1 to filopodia tips where integrin-adhesions enhance the stability of these structures and drive cancer cell invasion (Arjonen et al., 2014; Zhang et al., 2004). Thus, integrin traffic and filopodia formation appear to be linked and this will be an interesting topic for investigation in the future.

As described above, mutant p53 proteins promote invasion, in part, by enhancing RCP-dependent α5β1 integrin recycling. In addition, p53 mutants have been implicated in the recycling of MET, the HGF receptor, leading to enhanced MET signalling, invasion and cell scattering in response to HGF in a MET- and RCP-dependent manner (Muller et al., 2013). Tensin-4, an oncoprotein and a known β1-integrin-binding partner, provides an additional link between MET and β1 integrin traffic. Tensin-4 interacts with active MET, possibly coupling MET and β1 integrin, and stabilizes MET by inhibiting its endocytosis. Thus, the tensin-4–MET complex promotes cell survival, proliferation, tumour growth and cell migration (Muharram et al., 2014). It is therefore possible that the trafficking pathways of MET and β1 integrin are intimately linked and that distinct growth factor signals determine both endocytosis and the RCP recycling partner of integrins to mediate the appropriate cell behaviour.

## Conclusions and future perspectives

Major advances have been made in our understanding of how integrin traffic is regulated as well as its biological importance. An increasing number of studies have linked integrin endosomal traffic, and, in particular, its recycling to invasion and metastasis in cancer. Cross-talk between integrins and receptor tyrosine kinases is hard-wired into this system and will be an interesting area of research in the future as the details of growth-factor-induced regulation of integrin traffic under different conditions remains incompletely understood. However, it is clear that endosomal adaptors and scaffold proteins are key mediators of the coordinated traffic of these receptors and, interestingly, some of these adaptors, such as RCP or tensin-4, are upregulated in carcinomas (Muharram et al., 2014; Zhang et al., 2009). Furthermore, regulation of the activity of the Rho family of small GTPases, including Rac1 and RhoA, which are crucial downstream effectors of integrin signalling, has been linked to integrin traffic and is a key factor in determining cell shape, mobility and invasive capacity in many different cancer types (Jacquemet et al., 2013; Mai et al., 2014). Several studies have now established important regulatory steps in integrin traffic that determine whether the receptor is recycled or targeted for degradation. It appears that, unless the integrin is diverted towards recycling by proteins including WASH, SNX17 or CLIC3, the receptor is instead trafficked to lysosomes resulting in reduced integrin levels in cells. This suggests that upon inhibition of recycling degradation might become the default pathway of endocytosed integrin cargo.

Although many of the mechanistic details of integrin traffic are becoming increasingly well established, it is only poorly understood whether these pathways have any specificity towards certain integrin heterodimers. Distinct integrin heterodimers are known to exhibit tissue-specific expression and elicit specific signalling processes following ligand engagement. However, most of the currently established integrin traffic mechanisms are shared, for example, by all β1 integrins. How specificity is established and what are the mechanisms that allow cells to specifically regulate the traffic of a subset of ligand-engaged integrins is one of the major unanswered questions in the field.

In the field of cancer, metabolism is widely studied for its important role in regulating oncogenic proliferation and cancer cell survival. Interestingly, recent evidence has linked metabolism and glycolysis to the regulation of angiogenesis. Increased glycolysis in the sprouting endothelial tip cells is important for the cell's migratory properties and the glycolytic enzymes appear to localize to the cell leading edge with actin (De Bock et al., 2013). Furthermore, integrins have recently been shown to turn over by autophagy. In breast cancer cells, autophagy modulates cell migration and β1 integrin membrane recycling, suggesting a link between integrin traffic and metabolic alterations in cancer (Tuloup-Minguez et al., 2013). The potential links between metabolism and integrin traffic should be investigated in the future. This opens up the exciting possibility that integrin traffic could be regulating invasion and migration differentially in cancer cells and that the ‘Warburg effect’ might not only regulate cell proliferation but also invasion and metastasis through integrins.

**Box 1. Canonical integrin trafficking**

Integrin endocytosis can occur by several routes broadly classified as clathrin dependent and clathrin independent (see figure). Newly identified pathways include macropinocytosis from circular dorsal ruffles (CDRs) (Gu et al., 2011), F-actin-rich membrane projections on the apical cell surface, and a RhoA-dependent form of clathrin-mediated endocytosis (Mai et al., 2014), both triggered by growth factor receptor signalling. In addition, β1 integrin can be endocytosed via clathrin-independent carriers. In contrast to other endocytic routes, this glycosphingolipid- and actin-dependent pathway is initiated at the extracellular surface of the cell. Here, the carbohydrate-binding protein galectin-3 (indicated by the yellow star in the figure) interacts with the glycosylated extracellular domain of β1 integrin and has been suggested to promote mechanical deformation of the plasma membrane and clathrin-independent receptor endocytosis (Lakshminarayan et al., 2014).

Integrin recycling back to the plasma membrane occurs through one of two spatially and temporally distinct mechanisms often referred to as short-loop and long-loop pathways (Caswell and Norman, 2006; Morgan et al., 2009; Scita and Di Fiore, 2010). Recycling through the short-loop pathway is Rab4 dependent and promotes rapid delivery of receptors back to the plasma membrane. Alternatively, receptors entering the long-loop pathway relocate to Rab11-positive perinuclear recycling compartments (PNRC) prior to returning to the cell surface. These recycling pathways facilitate adhesion turnover and provide the cell with a constant fresh pool of integrins to engage the matrix and generate new adhesions.